# Differentially Methylated DNA Regions and Left Ventricular Hypertrophy in African Americans: A HyperGEN Study

**DOI:** 10.3390/genes13101700

**Published:** 2022-09-22

**Authors:** Alana C. Jones, Amit Patki, Steven A. Claas, Hemant K. Tiwari, Ninad S. Chaudhary, Devin M. Absher, Leslie A. Lange, Ethan M. Lange, Wei Zhao, Scott M. Ratliff, Sharon L. R. Kardia, Jennifer A. Smith, Marguerite R. Irvin, Donna K. Arnett

**Affiliations:** 1 Department of Epidemiology, School of Public Health, University of Alabama-Birmingham, Birmingham, AL 35233, USA; 2 Department of Biostatistics, School of Public Health, University of Alabama-Birmingham, Birmingham, AL 35233, USA; 3 Department of Epidemiology, College of Public Health, University of Kentucky, Lexington, KY 40506, USA; 4 Department of Epidemiology, Human Genetics, and Environmental Sciences, School of Public Health, University of Texas Health Science Center, Houston, TX 77030, USA; 5 Hudson Alpha Institute of Biotechnology, Huntsville, AL 35806, USA; 6 Department of Epidemiology, School of Public Health, University of Colorado, Aurora, CO 80045, USA; 7 Department of Biomedical Informatics, School of Medicine, University of Colorado, Aurora, CO 80045, USA; 8 Department of Biostatistics and Informatics, School of Public Health, University of Colorado, Aurora, CO 80045, USA; 9 Department of Epidemiology, School of Public Health, University of Michigan, Ann Arbor, MI 48109, USA

**Keywords:** epigenetics, DNA methylation, left ventricular hypertrophy, DMR, EWAS

## Abstract

Left ventricular (LV) hypertrophy (LVH) is an independent risk factor for cardiovascular disease, and African Americans experience a disparate high risk of LVH. Genetic studies have identified potential candidate genes and variants related to the condition. Epigenetic modifications may continue to help unravel disease mechanisms. We used methylation and echocardiography data from 636 African Americans selected from the Hypertension Genetic Epidemiology Network (HyperGEN) to identify differentially methylated regions (DMRs) associated with LVH. DNA extracted from whole blood was assayed on Illumina Methyl450 arrays. We fit linear mixed models to examine associations between co-methylated regions and LV traits, and we then conducted single CpG analyses within significant DMRs. We identified associations between DMRs and ejection fraction (*XKR6*), LV internal diastolic dimension (*TRAK1*), LV mass index (*GSE1*, *RPS15 A*, *PSMD7*), and relative wall thickness (*DNHD1*). In single CpG analysis, CpG sites annotated to *TRAK1* and *DNHD1* were significant. These CpGs were not associated with LV traits in replication cohorts but the direction of effect for *DNHD1* was consistent across cohorts. Of note, *DNHD1*, *GSE1*, and *PSMD7* may contribute to cardiac structural function. Future studies should evaluate relationships between regional DNA methylation patterns and the development of LVH.

## 1. Introduction

Left ventricular (LV) hypertrophy (LVH) is a thickening of walls of the left ventricle of the heart. This condition can cause the heart chamber to lose elasticity and may hinder relaxation of the LV during diastole, resulting in diastolic dysfunction. LVH is a common condition in the US, and it is more prevalent in African American (AA) adults than European-American (EA) adults [1,2]. In the Hypertension Genetic Epidemiology Network (HyperGEN) population, AAs had 1.80 higher odds of LVH (95% CI 1.29 to 2.51) than EA participants. [3]. In a separate cohort, the prevalence of LVH in AAs ranged from ~15% among non-hypertensive males to ~85% among females with obesity [4]. LVH is an independent risk factor for cardiovascular endpoints and has been associated with increased coronary artery disease, heart failure (HF), arrhythmia, stroke, and all-cause mortality [5,6,7,8,9,10,11,12,13,14].

Heritability estimates for LV mass—an echocardiography trait used in the clinical diagnosis of LVH—are high in AAs, e.g., 0.46 among HyperGEN offspring [15]. Some studies have reported higher heritability in AA versus EA populations [1]. Moreover, the heritability of other structural and functional echocardiographic phenotypes, such as relative wall thickness, LV internal dimension, and mitral annular velocities, are similarly high across multiple ancestry groups [16,17]. Additionally, a higher proportion of African ancestry has been positively associated with LVH [18]. Linkage [19,20,21], candidate gene association [20,22,23,24], and genome-wide association studies (GWAS) [25,26,27] of LVH and these echocardiographic phenotypes have reported suggestive and statistically significant findings in biologically relevant gene regions. Although there has been some success in identifying genetic variants associated with differences in LV phenotypes [20,24], there remains a large portion of unexplained heritability, particularly in AAs. Furthermore, although LV mass is largely similar across racial groups in early childhood, racial differences have been observed in adolescent populations, which may suggest a potential role for the environment in the development of LVH [28,29].

Epigenetic processes may explain a component of this unaccounted genetic variability, or “missing heritability” [30]. LVH is characterized by increased fibrosis and cardiomyocyte growth and may be a maladaptive response to stress from long-standing hypertension and other disorders. Structural and/or functional LV changes may be due to or trigger epigenetic processes such as DNA methylation and histone modifications that alter chromatin structure and affect cardiac gene expression [31]. For example, increased global genomic DNA methylation and changes in the expression of proteins in the LV have been observed in male adult rats with norepinephrine-induced cardiac hypertrophy [32].

The Identification of DNA methylation patterns associated with LVH and other LV phenotypes may facilitate new approaches for improved detection, treatment, and prognosis. Such discoveries remain a priority as LVH is an important predictor of HF in AAs and it is predicted that the US prevalence of HF will increase by 46% from 2012 to 2030 with total (direct and indirect) costs increasing from $31 billion to $70 billion [33,34]. The HyperGEN study of LVH was designed to detect genomic contributors to LV mass and related echocardiographic phenotypes using sibships ascertained on hypertension and their family members. In the current study, we evaluated the association of genome-wide markers of DNA methylation with these phenotypes in ~600 AAs from HyperGEN with validation in the Jackson Heart Study (JHS) and Genetic Epidemiology Network of Arteriopathy (GENOA).

## 2. Materials and Methods

### 2.1. Study Population

HyperGEN is a cross-sectional study and a component of the NHLBI Family Blood Pressure Program designed to identify genetic risk factors for hypertension and target end-organ damage due to hypertension [35]. The cohort is composed of EA and AA sibships in which at least two siblings were diagnosed with hypertension before age 60 years, their unmedicated adult offspring, and age-matched controls. Later, the study population was expanded to include other siblings of the original sibling pair, as well as any offspring for a total sample size of *n*~5000. For the echocardiography study, participants were recruited from the NHLBI Family Heart Centers (Atherosclerosis Risk in Communities (ARIC) Study and Utah Health Family Tree Study); Forsythe County, NC, USA; and Birmingham, AL, USA. Hypertension was defined as either self-reported use of antihypertensive medications or an average systolic blood pressure ≥ 140 mmHg and/or diastolic blood pressure ≥ 90 mmHg at two separate evaluations. For LV measurements, Doppler, two-dimensional (2D), and M-mode (2D-guided) echocardiograms were performed following a standardized protocol previously described [36]. Race was self-reported. This epigenetic study was conducted under an extreme phenotype sampling design, using data from 636 AA adults comprising the highest and lowest quartiles of LV mass indexed to height in meters (LVMHT27) [37,38]. The family structure of this study population is presented in Table S1.

### 2.2. Epigenome Analysis

DNA (500 ng) extracted from buffy coat was hybridized to the Illumina Infinium HumanMethylation450 BeadChip (450 K) array to assess methylation at cytosine-phosphate-guanine (CpG) sites. Analysis of the intensity files with Illumina GenomeStudio generated β scores of the proportion of signaling of the methylation probe in the sample and respective *p*-values. Quality control (QC) procedures removed CpG β scores with an association detection *p*-value greater than 0.01, samples with more than 1.5% missing data points, and any CpG probe where more than 10% of samples failed to yield adequate intensity. After these QC filters, there were 473,864 CpG sites eligible for analysis for 611 participants. For correction of systematic technical biases in the 450 K array, normalization was performed using the “noob” function from the R package *SeSAMe*, which uses a normal-exponential deconvolution method [39]. Cell count proportions (CD8^+^ T lymphocytes, CD4^+^ T lymphocytes, natural killer (NK) cells, B cells, monocytes, and granulocytes) were created using the algorithm developed by Houseman et al., which predicts underlying cellular composition of each sample from DNA methylation patterns [40].

### 2.3. Covariates

Covariates obtained at baseline included age; sex; body mass index (BMI); recruitment center; estimated proportions of CD8^+^ T lymphocytes, CD4^+^ T lymphocytes, NK cells, B cells, and monocytes (granulocytes as the reference) [40]; the first four principal components (PCs) of ancestry generated from GWAS data as previously described [41]; and batch. Participant ID was a random effect. Age and sex were self-reported. BMI was calculated as a ratio of measured weight (kg) to square of height (m^2^).

### 2.4. Statistical Analysis

We used the *coMethDMR* package in R version 4.0.3 (12 October 2020) to detect associations between differentially methylated DNA regions (DMRs) and traits related to LVH: LVMHT27, midwall shortening (MWS), relative wall thickness (RWT), left atrial systolic dimension (LASD), left ventricular internal diastolic dimension (LVIDD), and ejection fraction (EF) [42]. First, we identified co-methylated regions independent of any outcome using the “coMethAllRegions” function. This method used a pre-defined list of clusters of at least three contiguous CpG sites in which the maximum separation between any two consecutive probes on the 450 K array was 200 bp and extracted regions in which CpG M-values were correlated with the sum of methylation within that region (*r* > 0.4). In HyperGEN, there were 29,327 regions that met these criteria. We then conducted linear mixed regression models using the “lmmTestAllRegions” function to determine associations between co-methylated regions (outcome) and LV traits (predictor). Models were adjusted for the covariates listed above, and regions with a false discovery rate (FDR) < 0.1 are reported.

As a sensitivity analysis, we also conducted an epigenome-wide association study (EWAS) to uncover independent effects of individual CpG sites (outcomes) within and outside significant DMRs. Linear mixed models were adjusted for age, sex, BMI, ancestry PCs, and cell counts as fixed effects and batch and family relatedness as random effects. CpG analyses were conducted using the *lmer* package, and FDRs were calculated from model *p*-values using the *stats* package, all in R. Results with FDR < 0.1 were considered statistically significant. We further evaluated the effect of relatedness in HyperGEN by replicating the main DMR analysis for our top findings using the residuals of CpG β scores adjusted for family ID and/or batch as random effects.

### 2.5. Replication

JHS is a prospective population-based study initiated to seek the causes of the high prevalence of common complex diseases among AAs in the Jackson, MS, metropolitan area [43]. Three exam cycles were as follows: 2000–2004 (visit 1), 2005–2008 (visit 2), and 2009–2012 (visit 3). DNA extracted from whole blood collected from the first visit was assayed with the Illumina EPIC 850 K array. Ancestry PCs were generated from GWAS array data as previously described [44]. Two-dimensional directed M-mode and Doppler echocardiographic data were collected using the same methods described in HyperGEN [36]. A total of 1054 participants from 831 distinct family clusters contributed phenotype and epigenotype data to the current analysis. Due to differences between the 450 K (HyperGEN) and EPIC (JHS) arrays, we excluded non-overlapping CpG sites between the arrays. We selected only CpG sites from co-methylated regions in HyperGEN for replication in JHS. Therefore, we did not determine co-methylated regions in JHS independently. Parallel models were used to test the associations between both co-methylated regions and individual CpG sites and LV traits (LVMHT27, RWT, and EF). Measurements for LASD, LVIDD, and MWS were not available for JHS. Replication models were adjusted for the same covariates as the discovery models except recruitment center, which was not applicable for JHS. Finally, linear mixed models were fit to test for associations between the β scores of single CpG sites within significant DMRs and LV traits, adjusting for the same covariates as the discovery model.

JHS was the primary replication cohort, but we further validated single CpG associations from the discovery analysis (within DMRs) in GENOA using parallel models as in JHS, with additional adjustment for time between blood collection and echocardiography measurement. GENOA consists of EA and AA hypertensive sibships that were recruited for linkage and association studies to identify genes that influence blood pressure and its target organ damage [45]. In Phase I (1996–2001), members of sibships containing ≥2 individuals with essential hypertension clinically diagnosed before age 60, including both hypertensive and normotensive siblings were invited to participate; of these, 1854 AAs were recruited from Jackson, MS. In Phase II (2000–2004), 1482 AA participants were successfully re-recruited to measure potential target organ damage due to hypertension. Due to overlaps in participants between JHS and GENOA, GENOA participants that were also in JHS were excluded. A total of 839 AA GENOA participants contributed phenotype and epigenotype data to the current analysis. Both HyperGEN and GENOA had identical protocols for the measurement of blood pressure, definition of hypertension, and echocardiography. DNA extracted from whole blood was assayed using the Illumina EPIC array at the first study visit.

## 3. Results

Characteristics of the discovery and replication cohorts are described in Table 1. The distribution of males and females, as well as BMI, was similar across the three cohorts. GENOA participants had the highest mean systolic and diastolic blood pressures and were on average older than participants in HyperGEN and JHS. As expected, JHS (19.4%) had a much lower prevalence of hypertension than HyperGEN (75.1%) and GENOA (78.7%). HyperGEN participants had the highest average LV mass, whereas RWT and EF were comparable across all three cohorts.

**Figure 1:**
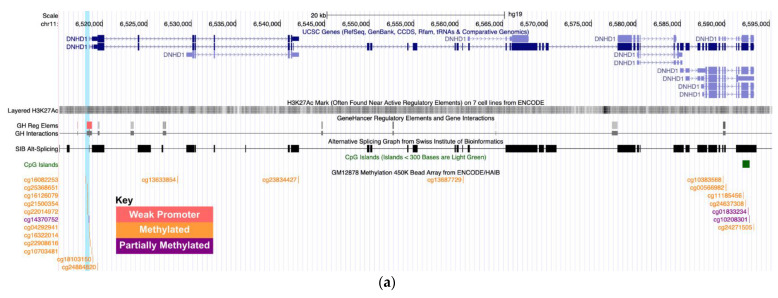

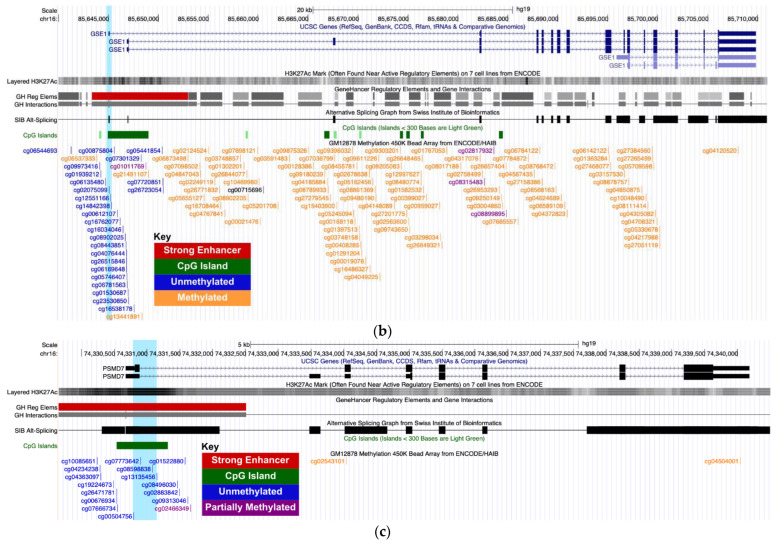
Biological features near (**a**) *DNHD1*, (**b**) *GSE1*, and (**c**) *PSMD7* from ENCODE. Highlighted fragments correspond to the DMR position.

In HyperGEN, we identified six DMRs that were associated with four of six measured LVH-related traits with FDR < 0.1 (Table 2). There was a positive association between methylation in the XK-Related Protein 6 (*XKR6*) region and EF, as well as between Trafficking Kinesin Protein 1 (*TRAK1*) and LVIDD. Conversely, there was an inverse relationship between DMRs at Genetic Suppressor Element 1 (*GSE1*), Ribosomal Protein S15a (*RPS15A*), and Protease 26S Subunit, Non-ATPase 7 (*PSMD7*) with LVMHT27, as well as between Dynein Heavy Chain Domain 1 (*DNHD1*) and RWT. DMRs that were associated with LVMHT27 were all located at CpG islands, but the DMRs linked to EF, LVIDD, and RWT were in open sea regions. We did not detect any significant associations with LASD or MWS. Figure 1 shows the DMRs for *DNHD1*, *GSE1*, and *PSMD7*. In JHS, these DMRs were not significantly associated with their respective LV traits. Single CpG association results for sites within significant DMRs are presented in Table 3. Among these, one CpG site near *TRAK1* and two CpG sites near *DNHD1* were statistically significant in HyperGEN. In JHS and GENOA, the directions of the associations were consistent for *DNHD1*, but they were not statistically significant.

**Figure 2:**
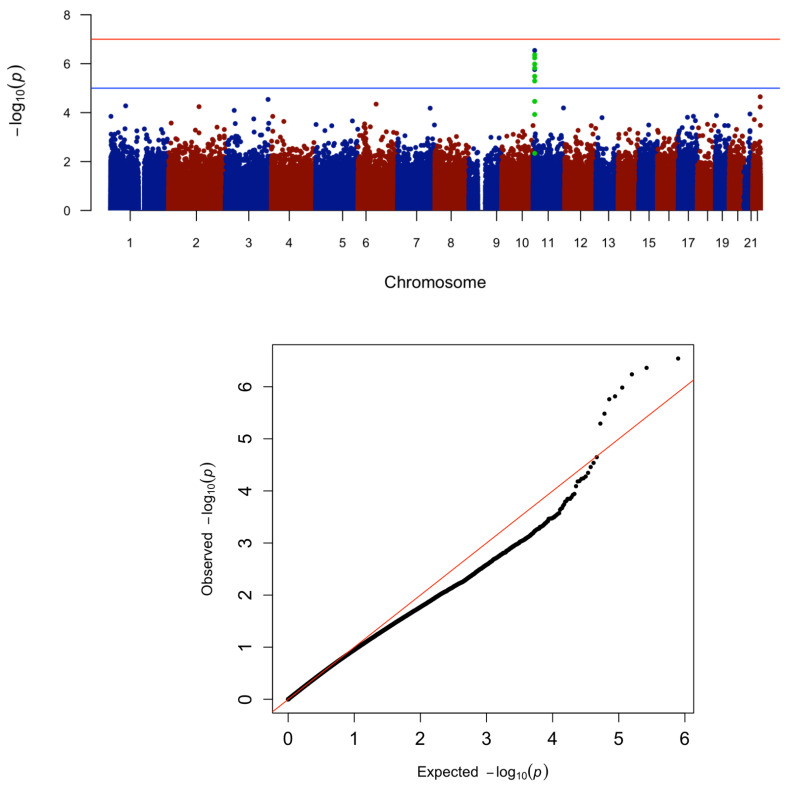
Manhattan & QQ Plot for RWT in HyperGEN. CpG sites in the *DNHD1* DMR are highlighted in green. Lambda with genomic control for QQ plot = 0.998.

Selected CpG EWAS results from HyperGEN (FDR < 0.1) are presented in Table S2. We identified three CpG sites annotated to *DNHD1*—two of which were in the significant DMR—in association with RWT (Figure 2). We also discovered additional CpG sites strongly associated with LASD near G Protein Subunit α 11 (*GNA11*) that were not identified by the primary DMR analysis. LASD data was not available in JHS, and these CpG sites were not replicated in GENOA. Furthermore, *DNHD1* associations remained robust in residuals-based sensitivity analyses (Table S3).

## 4. Discussion

There is a need to better understand susceptibility to LVH in high-risk groups to create targeted prevention and therapeutic strategies. Previous GWAS have identified genetic variants associated with LVH in multiple ethnic groups [20,46,47,48]. However, the role of the epigenome in LVH has not been fully explained. We found significant associations between DNA methylation (both at the regional and individual CpG level) and LV traits in a cohort of AAs enriched for hypertension. Although these regions did not meet the required significance threshold for replication in the validation cohorts, further investigation remains warranted to better understand the potential role of DNA methylation in LVH, particularly in AAs, who experience this condition 2–3 times more frequently than EAs [2].

Of our top findings, three gene regions—*DNHD1*, *GSE1*, and *PSMD7*—had biological and statistical support for the observed associations. Dynein heavy chain domain 1 (*DNHD1*) encodes a protein that is involved in cellular microtubule motor activity. Recent studies indicate that this gene may influence vascular structural integrity, and may contribute to in congenital heart defects [49,50]. Additionally, *DNHD1* methylation has been linked to prenatal lead exposure [51]. These findings suggest that *DNHD1* expression may be associated with cardiac pathology, and environmental exposures may alter its methylation. In our study, we observed that decreased methylation of a nine-CpG region near the *DNHD1* promoter was associated with increased relative wall thickness (Figure 1a). Although the association was not replicated, we observed consistent directions of effect among individual CpG associations in both JHS and GENOA. Further investigation of *DNHD1* methylation is warranted to understand its role in modulating relative wall thickness and LVH.

Genetic suppressor element 1 (*GSE1*) encodes a subunit of a histone deacetylase complex, and it has been shown to be highly expressed in epicardial adipose tissue (EAT), a correlate of LV mass [52]. We identified a DMR at *GSE1* that was inversely related to LV mass index. This region overlaps with an enhancer-like signature (Figure 1b); further, a CpG in this region (cg14842398) has been linked to insulin resistance, which may promote LVH development [53,54,55,56,57]. Similarly, we identified a DMR at *PSMD7* with an inverse association with LV mass index (Figure 1c). This gene codes for a subunit of the 26S proteasome complex, which is involved in protein homeostasis. In a gene network analysis, *PSMD7* was upregulated in hypertensive patients with LV remodeling compared to those without LV remodeling [58]. Like *GSE1*, the *PSMD7* DMR has strong enhancer activity and is an alternative splicing site. More studies are needed to characterize the relationship between methylation variations of these genes and LV-related traits.

Our study is among the first to report variations in DNA methylation in association with LVH in humans and the first in African Americans. *DNHD1*, *GSE1*, and *PSMD7* may be genes of functional significance. While our findings were not replicated in JHS or GENOA with statistical significance, we observed similar directions of association, and there was biological plausibility for the observed results. Notably, *DNHD1* associations persisted in multiple sensitivity analyses (Tables S2 and S3). Furthermore, *DNHD1*, *GSE1*, and *PSMD7* have previously been linked to cardiac structural changes and/or risk factors for LVH. Overall, more studies are needed to clarify the relationship between methylation and expression of these genes, as well as the mechanisms by which they influence LVH pathophysiology.

Strengths of our study include our large sample size of AAs with echocardiography and epigenomic data (total *n*~2500), as well as the incorporation of two external cohorts for validation of our findings. HyperGEN, although smaller than JHS and GENOA, is unique in that it leveraged an extreme phenotype sampling design, which has been shown to increase study power in genetic association studies [38]. Because the *coMethDMR* package is still a relatively novel method of evaluating DMRs, we also confirmed our findings in EWAS and detected similar CpG-level associations. These results demonstrate that regional level analyses can highlight methylation variations associated with disease when single CpG analyses may be underpowered to detect these differences.

Limitations include the cross-sectional design of HyperGEN, as we were not able to ascertain causality of these findings. HyperGEN participants and the replication cohorts were also epigenotyped on different arrays, but this limitation is lessened by an approximately 90% overlap of the Illumina 450 K array CpG sites with those on the EPIC array [59]. Findings were not replicated with statistical significance, but potential differences in age and environmental exposures may account for differences in methylation patterns between HyperGEN and the replication cohorts.

In conclusion, this study evaluated the role of DNA methylation in LVH and LV phenotypes in African Americans in attempt to address the lack of epigenetic research focused on this condition in this population. Our top results were focused on genes related to vascular structure (*DNHD1*), epigenomic regulation (*GSE1*) and protein degradation (*PSMD7*). Differences in the study populations with respect to age or the environment may have hindered statistical replication of our results. Additional validation, especially for *DNHD1*, is still needed to determine the potential utility of these findings with respect to prevention and/or treatment of LVH.

## Figures and Tables

**Table 1:** Characteristics of African Americans in HyperGEN, JHS, and GENOA.

N (%)/Mean (SD)	HyperGEN (N = 611)	JHS (N = 1054)	GENOA (N = 839)
Age, years	48.4 (11.1)	53.7 (12.1)	62.7 (9.6)
Male	205 (33.4%)	378 (35.9%)	242 (28.8%)
Body mass index, kg/m^2^	32.4 (8.2)	32.0 (7.2)	31.4 (6.6)
Systolic blood pressure, mmHg	131.6 (23.6)	126.7 (16.1)	143.2 (23.4)
Diastolic blood pressure, mmHg	75.5 (12.6)	76.2 (8.5)	79.3 (11.5)
Hypertension, %	459 (75.1%)	205 (19.4%)	660 (78.7%)
LV mass, g (LVM)	180.0 (63.3)	151.6 (41.1)	160.1 (48.6)
LV mass indexed to height, g/m (LVMHT27)	45.0 (16.1)	36.9 (9.8)	39.3 (11.3)
Relative wall thickness, cm (RWT)	0.3 (0.1)	0.4 (0.1)	0.3 (0.1)
LV internal diastolic dimension, cm (LVIDD)	5.2 (0.6)	-	5.2 (0.5)
Left atrial systolic dimension, cm (LASD)	3.4 (0.6)	-	3.6 (0.5)
Ejection fraction, % (EF)	61.8 (6.9)	62.1 (7.8)	60.6 (7.7)
Midwall shortening, % (MWS)	17.0 (2.7)	-	17.6 (2.4)

LV: left ventricular.

**Table 2:** Statistically significant DMRs by LV trait.

Trait	Chr	nCpGs	DMR (bp)	Location	Gene	HyperGEN		JHS	
						β(SE)	FDR	β(SE)	FDR
EF	8	3	59	Open Sea	*XKR6*	0.0048(0.0012)	**0.056**	0.0010(0.0013)	1
LVIDD	3	5	335	Open Sea	*TRAK1*	0.1130(0.0270)	**0.042**	--	--
LVMHT27	16	3	247	Island	*GSE1*	−0.0021(0.0005)	**0.085**	0.0003(0.0005)	0.61
	16	5	225	Island	*RPS15 A*	−0.0018(0.0005)	**0.085**	−0.0001(0.0005)	0.75
	16	4	350	Island	*PSMD7*	−0.0026(0.0007)	**0.085**	−0.0012(0.0012)	1
RWT	11	9	469	Open Sea	*DNHD1*	−1.2688(0.2749)	**0.008**	−0.1598(0.1781)	0.37

Chr: chromosome. FDR: false discovery rate. LVIDD measurements were not available in JHS participants. Linear mixed models were adjusted for age, sex, BMI, cell counts, ancestry principal components, batch, and sample ID (random). There were no significant (FDR < 0.1) DMRs associated with LASD or MWS in HyperGEN. Models with FDR = 1 did not converge.

**Table 3:** CpG associations LV traits within significant DMRs in HyperGEN.

Trait	Chr	Gene	CpG	HyperGEN		JHS		GENOA	
				β	FDR	β	FDR	β	FDR
EF	8	*XKR6*	cg27534424	0.0002	1	1.18 × 10^−4^	0.17	1.24 × 10^−5^	0.89
			cg09051192	0.0001	1	7.01 × 10^−5^	0.23	3.69 × 10^−5^	0.80
			cg23673360	9.76 × 10^−5^	1	7.26 × 10^−5^	0.19	−5.62 × 10^−6^	0.89
LVIDD	3	*TRAK1*	cg01855070	0.013	0.83	--	--	−0.0038	0.89
			cg08804892	0.011	1	--	--	−0.0008	0.89
			cg08508763	0.015	0.93	--	--	−0.0037	0.89
			**cg23715029**	0.023	**0.06**	--	--	−0.0017	0.89
			cg03168947	0.019	0.32	--	--	−0.0043	0.89
LVMHT27	16	*GSE1*	cg02075099	−4.60 × 10^−5^	1	6.43 × 10^−6^	0.58	1.39 × 10^−6^	0.89
			cg12551166	−7.62 × 10^−5^	1	3.10 × 10^−5^	0.20	1.39 × 10^−5^	0.80
			cg14842398	−4.36 × 10^−5^	1	−1.49 × 10^−5^	0.58	−1.24 × 10^−5^	0.89
		*RPS15 A*	cg07623022	−1.51 × 10^−5^	1	7.73 × 10^−6^	0.58	−1.92 × 10^−5^	0.80
			cg01527529	−6.52 × 10^−5^	1	−2.72 × 10^−5^	0.41	−4.77 × 10^−6^	0.89
			cg21024145	−4.42 × 10^−5^	1	3.71 × 10^−5^	0.32	4.50 × 10^−6^	0.89
			cg26593997	−7.54 × 10^−5^	1	6.52 × 10^−7^	1	4.92 × 10^−6^	0.89
			cg01680999	−4.76 × 10^−5^	1	−1.72 × 10^−5^	0.56	−9.40 × 10^−6^	0.89
		*PSMD7*	cg00504756	−7.08 × 10^−5^	1	1.99 × 10^−5^	0.52	4.82 × 10^−5^	0.80
			cg07773642	−4.86 × 10^−5^	1	2.36 × 10^−5^	0.72	2.08 × 10^−6^	0.89
			cg13135456	−5.94 × 10^−5^	1	−2.26 × 10^−5^	0.37	−3.99 × 10^−5^	0.80
			cg08598838	−3.14 × 10^−5^	1	−3.32 × 10^−5^	0.19	−3.67 × 10^−6^	0.89
RWT	11	*DNHD1*	cg14370752	−0.156	1	--	--	−0.0138	0.89
			cg16322014	−0.131	0.25	−0.016	0.37	−0.0020	0.89
			**cg22908616**	−0.153	**0.08**	−0.052	0.17	−0.0309	0.80
			cg16082253	−0.079	1	0.021	0.32	0.0040	0.89
			cg25368651	−0.110	0.10	−0.031	0.19	−0.0102	0.80
			**cg16126079**	−0.121	**0.08**	−0.026	0.21	−0.0044	0.89
			cg21500354	−0.139	0.11	−0.027	0.33	−0.0141	0.80
			cg22014972	−0.127	0.19	−0.035	0.19	−0.0116	0.80
			cg04292941	−0.083	1	−0.021	0.52	−0.0144	0.89

Chr: chromosome. ‘--’ indicates that either CpG or LV trait data were not available. Linear mixed models were adjusted for age, sex, BMI, cell counts, ancestry principal components, family relatedness (random), and batch (random). GENOA models were further adjusted for time between blood sample collection and echocardiography measurement. Boldface indicates statistical significance (FDR < 0.1). There were no significant DMRs associated with LASD or MWS in HyperGEN.

## Data Availability

The datasets used and/or analyzed during the current study are not publicly available. HyperGEN data are available from the corresponding author upon reasonable request. The corresponding author will assist requesters with acquiring relevant data from the JHS and GENOA coordinating sites.

## Supplementary Materials

The following are available online at: https://www.mdpi.com/article/10.3390/genes13101700/s1, Table S1. HyperGEN EWAS Family Structure, Table S2. Selected EWAS results from HyperGEN, Table S3. Association of DNHD1 DMR with RWT in HyperGEN using family and batch-adjusted residuals.
