# Supplementary material for: Differentially Methylated DNA Regions and Left Ventricular Hypertrophy in African Americans: A HyperGEN Study
Source: Genes (Basel). 2022 Sep 22;13(10):1700. doi: 10.3390/genes13101700 (PMC9601679; doi:10.3390/genes13101700)
Supplement: Supplementary file 1 [file genes-13-01700-s001.zip › genes-1811148-supplementary.pdf]

**Table S1. HyperGEN EWAS Family Structure**					
	**Family Size**				
	**1**	**2**	**3**	**4**	**5**
Members per family	334	82	30	4	2
Parent-child pairs	46				
Full sibling pairs	42				

EWAS: epigenome-wide association study. Members per family describes the number of distinct family clusters. For example, for Family Size = 2, there are 82 families with 2 members (82 x 2 = 164 total participants).

Table S2. Selected EWAS results from HyperGEN.

Trait	Chr	Gene	CpG	β	SE	FDR
LASD	19	*GNA11*	cg05370802	-0.0062	0.0009	5.36E-09
			cg18505103	-0.0017	0.0003	2.93E-06
	10	*PANK1*	cg00257187	-0.0101	0.0017	3.41E-06
	7		cg17512207	-0.0158	0.0027	8.13E-06
	15	*SLC24A1*	cg07116160	-0.0106	0.0020	1.28E-04
	14		cg05015996	-0.0088	0.0018	2.48E-03
	8	*ENTPD4*	cg04712321	0.0168	0.0037	0.020
	14	*KCNK10*	cg12095807	-0.0083	0.0018	0.020
	7		cg22886275	0.0135	0.0030	0.020
	5		cg01195564	-0.0160	0.0036	0.035
	1		cg09132102	-0.0163	0.0037	0.035
	6	*TRIM15*	cg13252226	-0.0161	0.0037	0.041
	14		cg18991417	0.0147	0.0034	0.041
	10		cg23939236	-0.0087	0.0020	0.044
	13		cg11508437	-0.0085	0.0020	0.071
	17	*MPRIP*	cg23842572	0.0119	0.0028	0.071
LVIDD	16	*VAC14*	cg00370608	0.0120	0.0025	0.056
	9	*NCRNA00092*	cg00462570	0.0169	0.0035	0.056
	3	*TRAK1*	**cg23715029**	0.0229	0.0049	0.056
	6	*JARID2*	cg26797722	0.0102	0.0022	0.056
MWS	6	*MAS1L*	cg07511633	0.0024	0.0004	0.012
		B3GAT2	cg10766373	-0.0006	0.0001	0.064
	17		cg05720491	0.0016	0.0003	0.064
	22		cg19115805	-0.0012	0.0003	0.064
RWT	11	*DNHD1*	cg10703481	-0.129	0.0224	0.076
			**cg22908616**	-0.1526	0.0270	0.076
			**cg16126079**	-0.1210	0.0216	0.076

EWAS results with FDR < 0.1. Chr: chromosome. Linear mixed models adjusted for age, sex, BMI, recruitment center, cell counts, ancestry principal components, family relatedness (random), and batch (random). Boldface indicates CpG was part of a significant DMR in main analysis. There were no significant CpG sites associated with EF.

Table S3. Association of *DNHD1* DMR with RWT in HyperGEN using family and batch-adjusted residuals.

Residuals	β(SE)	P
Family[#]	-0.1194(0.0238)	5.34e-07
Family & batch[##]	-0.1236(0.0223)	3.09e-08

[#]Family ID as a random effect. [##]Family ID and batch as random effects. Models adjusted for age, sex, BMI, recruitment center, ancestry principal components, cell counts, and batch (family-only residuals).
